# The Expression of Antibacterial Peptide Turgencin A in *Pichia pastoris* and an Analysis of Its Antibacterial Activity

**DOI:** 10.3390/molecules28145405

**Published:** 2023-07-14

**Authors:** Chunming Dong, Mengru Li, Rui Zhang, Weitao Lu, Lijun Xu, Jian Liu, Xinlei Chu

**Affiliations:** 1 College of Marine and Environmental Sciences, Tianjin University of Science and Technology, Tianjin 300457, China; lmrzst@163.com (M.L.); zhangrui9971@163.com (R.Z.); lwt190826@163.com (W.L.); xlj13363631028@163.com (L.X.); 2 College of Agriculture and Bioengineering, Heze University, Heze 274000, China; 3 Department of Epidemiology and Biostatistics, Tianjin Medical University Cancer Institute and Hospital, Tianjin Medical University, Tianjin 300060, China

**Keywords:** antimicrobial peptide, *Pichia pastoris*, *Staphylococcus aureus*, membrane damage, pork preservation

## Abstract

Antibiotic resistance to pathogenic bacteria is becoming an increasing public health threat, and identifying alternatives to antibiotics would be an effective solution to the problem of drug resistance. Antimicrobial peptides are small peptides produced by various organisms; they are considered to be adequate antibiotic substitutes because they have intense, broad−spectrum antibacterial activity and stability, are widely available, and target strains do not quickly develop resistance. Recent research on antimicrobial peptides has shown that they have broad potential for applications in medicine, agriculture, food, and animal feed. Turgencin A is a potent antimicrobial peptide isolated from the Arctic sea squirt. We established a His-tagged expression system for *Pichia pastoris* and developed a rTurgencin A using the recombinant expression in *Pichia pastoris* with nickel column purification. This antimicrobial peptide showed intense antimicrobial activity against Gram-positive and Gram-negative bacteria and a good stability at most temperatures and pHs, as well as in various protease and salt ion concentrations, but underwent a significant decrease in stability in high-temperature and low-pH environments. Turgencin A induced bacterial membrane rupture, resulting in content leakage and subsequent cell death. It was also shown to have low hemolytic activity. This study provides primary data for the industrial production and application of the antimicrobial peptide Turgencin A.

## 1. Introduction

Bacterial resistance due to the misuse of antibiotics is one of the biggest threats to global health, food security, and development [1], and the resistance of many antibiotic-sensitive bacteria is increasing [2,3]. *Staphylococcus aureus* (*S. aureus*) is a common foodborne microorganism widely found in the natural environment, where it produces enterotoxins that cause food poisoning [4], which accounts for approximately 25% of the food poisoning incidents related to foodborne microorganisms [5]. Antibiotics effectively inhibit the growth of *S. aureus*; however, the long-term use of large quantities of antibiotics increases bacterial resistance and residuals during treatment [6]. Therefore, it is crucial to identify new antimicrobial substances that can inhibit *S. aureus* infection.

Antimicrobial peptides are a class of small molecule peptides with broad-spectrum antimicrobial activity against bacteria, fungi, and viruses, which show sound antibacterial effects against Gram-negative and Gram-positive bacteria [7]. They consist of between 10 and 50 amino acids and are now candidates for addressing the challenges associated with antibiotic resistance [8,9]. Antimicrobial peptides are derived from a wide range of sources, and as many as 3000 have been identified and isolated from mammals [10], amphibians [11], marine animals [12], insects [13], and plants [14,15]. Antimicrobial peptides can rupture bacteria by forming ion channels in cell membranes [16], piercing pores, and promoting membrane disintegration. They can also enter cells to act on nucleic acids and affect the synthesis and replication of DNA and RNA, thus causing cell death [17,18,19,20,21]. Antimicrobial peptides are less likely to develop resistance than traditional antibiotics, and their antibacterial action is swifter [22]. They provide a safe and non-toxic effect, stability in highly acidic and alkaline environments, heat stability, broad-spectrum antibacterial properties, and antioxidant and free-radical-scavenging functions, which means that they can be widely used in medicine, food preservation, livestock breeding, and other industries [23,24,25].

Oceans cover 71% of the Earth’s surface and their organisms account for 50–80% of global biodiversity; therefore, marine organisms are a rich source of biological antimicrobial peptides [26,27]. Turgencin A consists of 36 amino acid residues and 3 disulfide bonds, and it is a potent antimicrobial peptide that has been isolated from the Arctic sea squirt *Synoicum turgens* [28]. Turgencins were the first sea squirt/periphyton to be isolated and characterized as being abundant in cysteine-rich antimicrobial peptides [29]. They have antibacterial activity against both Gram-negative and Gram-positive bacteria and are also highly stable at various pH levels and temperatures, which makes them a potential antibiotic alternative. However, *Synoicum turgens* contain short amounts of this antibacterial peptide, and as the isolation process is complicated, purification is more difficult and large-scale production cannot be achieved [30,31]. It is difficult to synthesize Turgencin A chemically [32], and the synthesized antimicrobial peptide does not adequately restore the secondary and tertiary structures of the original antimicrobial peptide, meaning the antimicrobial activity of this peptide is significantly reduced.

In this study, we constructed a *Pichia pastoris* strain for the genetically engineered production of the antagonistic peptide Turgincin A and successfully expressed the active Turgencin A in *Pichia pastoris*. The antibacterial activity of the recombinant peptide was higher in Gram-positive bacteria than in Gram-negative bacteria. The stability of the recombinant antimicrobial peptide Turgencin A was investigated using an inhibition circle assay, and it showed a good stability under most temperatures, pH levels, and protease treatments. However, its inhibition activity decreased under high-temperature and low-pH conditions. Propidium iodide staining and scanning electron microscopy (SEM) experiments showed that the recombinant antimicrobial peptide Turgencin A disrupted bacterial cell membranes, which resulted in bacterial death via leakage of the bacterial contents. We also confirmed via hemolysis assays that the antimicrobial peptide has low hemolytic activity. We also investigated the application of the antimicrobial peptide in food preservation. We found that the recombinant antimicrobial peptide Turgencin A inhibited the growth of the total bacteria in pork and maintained the color of the meat.

## 2. Results and Discussion

### 2.1. Structural Analysis of Recombinant Turgencin A Strains

**Figure 1:**
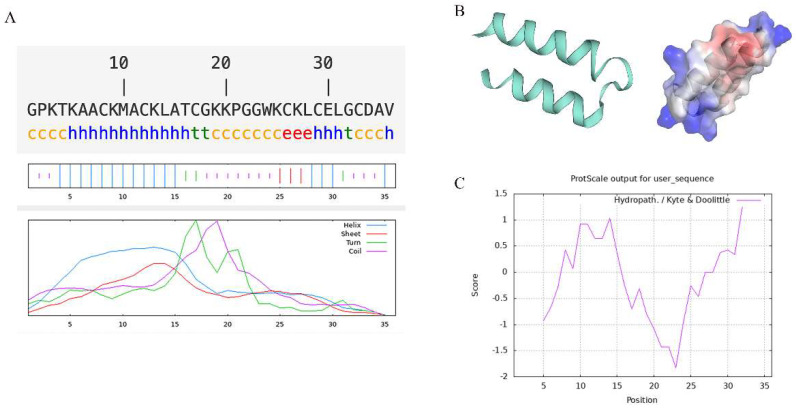
(**A**) secondary structure prediction. Alpha helix (h), Random coil (c), Extended strand (e), and Beta turn (t); (**B**) tertiary structure prediction and electrostatic potential analysis (blue area electrostatic potential value is positive and red area electrostatic potential value is negative); and (**C**) hydrophobicity analysis (hydrophilic regions are represented by negative values and hydrophobic regions are represented by positive values).

The secondary structure analysis showed that the main structure of Turgencin A is an alpha helix and random coil, accounting for 44.44% and 38.89%, respectively (Figure 1A). The α helix is the main structure of Turgencin A, which plays an essential role in its antibacterial activity [33]. The tertiary structure analysis showed the α helix and random coil structure of Turgencin A more intuitively. The electrostatic potential diagram showed that Turgencin A is a small cationic peptide (Figure 1B), which may interact with the cell membranes of negatively charged Gram-positive and Gram-negative bacteria, leading to bacterial cell membrane rupture and cell death [34]. Nevertheless, a positive charge does not seem essential, and the antibacterial activity of anionic peptides may be related to hydrophobic amino acid residues [35]. A hydrophobicity analysis showed that Turgencin A is an amphoteric amino acid but is hydrophilic, which benefits its adsorption on bacterial surfaces (Figure 1C). The structure prediction and hydrophobicity analysis of antimicrobial peptides provide some theoretical support for subsequent recombinant expression and antibacterial mechanism research [36].

### 2.2. Construction and Recombinant Expression of Positive Yeast Strains with High Expression of Recombinant Turgencin A

**Figure 2:**
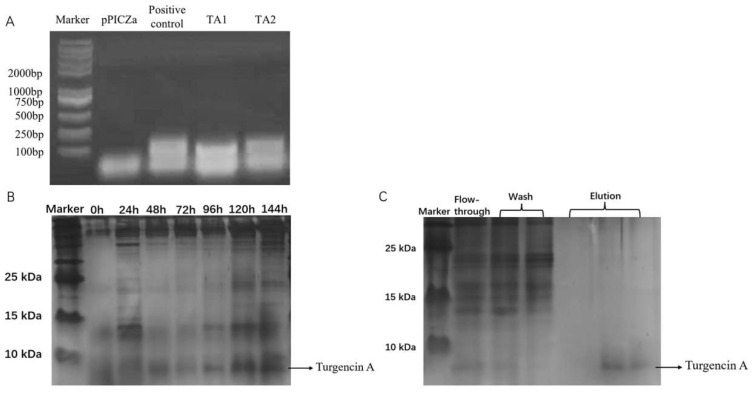
(**A**) PCR agar gel electrophoresis; the TA1 and TA2 in the figure are the transformed monoclonal yeast strains. The positive control is the synthesized plasmid containing Turgincin A and the negative control is a pPICZαA null load without the Turgencin A gene. (**B**) Expression of Turgencin A at different times; and (**C**) Ni purification of rTurgencin A. (Lane 1: flow-through solution, Lane 2–3: impurity protein wash solution, and Lane 4–6: target protein elution solution.).

The constructed pPICZαA-Turgencin A plasmid was transferred into *Pichia pastoris*. The recombinant yeast genomic DNA was extracted and the target fragment was amplified using PCR (Figure 2A). The TA1 and TA2 in Figure 2 are the transformed monoclonal yeast strains. The positive control is the synthesized plasmid containing Turgincin A, and the negative control is a pPICZαA empty vector. As shown in Figure 2, the PCR product of TA1 and TA2 was consistent with the positive control. To further verify this, the target band was recovered for DNA isolation and sent to Genewiz for DNA sequencing. It was found that the gene of the transformant contained the gene of Turgincin A, indicating that the target gene was successfully transferred into *Pichia pastoris* (Figure S1).

Positive yeast cells were subjected to an expression analysis and samples were taken every 24 h. The protein expression level was analyzed using Tricine sodium dodecyl sulfate-polyacrylamide gel electrophoresis (Tricine-SDS-PAGE). The electrophoretic gel plot (Figure 2B) showed that the amount of Turgencin A secreted by *Pichia pastoris* increased in the induction time from 0 h to 120 h. The molecular weight of the recombinant antimicrobial peptide was approximately 7 kDa. However, there was no significant increase in expression from 120 h to 144 h. This indicates that Turgencin A expression reaches its highest level at 120 h. The optimal time to induce Turgencin A expression was 120 h.

The fermentation supernatant was collected after the expression and was subjected to affinity purification using a nickel column (Figure 2C). Turgencin A was successfully eluted after rinsing. Due to the high concentration of imidazole in the eluate, which also has antibacterial effects, it was necessary to remove the imidazole in the Turgencin A eluted from the nickel column via dialysis. The concentrations of recombinant Turgencin A (rTurgencin A) in different batches of supernatants and lyophilized powder dilutions were measured using ELISA. The primary antibody used was Anti His-Tag Mouse MAb and the secondary antibody was HRP-Goat-anti-mouse. The results are shown in Table 1. Due to the slight differences in the experimental conditions and environment during each induction of expression, the content of recombinant antimicrobial peptide Turginin A produced after each induction of expression was also different. After the induction of expression in different batches, the concentration of the recombinant antimicrobial peptide Turginin A was obtained, with a concentration of 11.23 μg·mL^−1^. The concentration of the yeast fermentation supernatant of recombinant Turgincin A in a 200 mg·mL^−1^ freeze-dried powder solution was 13.71 μg·mL^−1^.

### 2.3. Determining Antibacterial Activity of Recombinant Turgencin A

**Figure 3:**
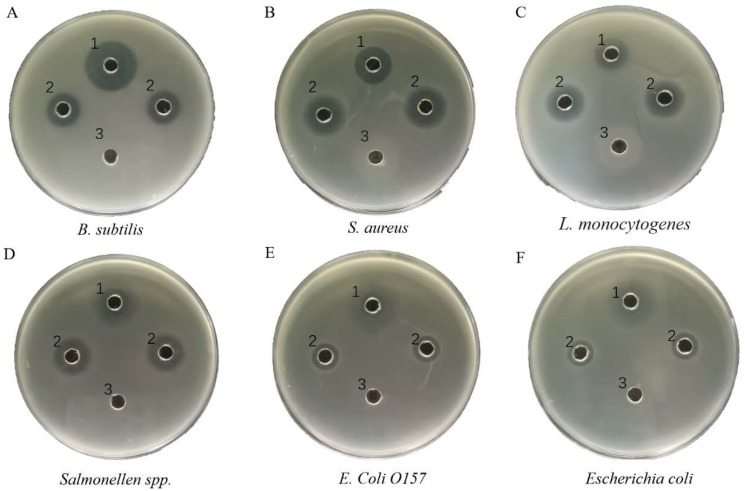
Bacteriostatic activity of Turgencin A: (**A**) *B. subtilis* ATCC6633; (**B**) *S. aureus* ATCC25923; (**C**) *L. monocytogenes* ATCC21633; (**D**) *Salmonella* spp. ATCC10467; (**E**) *E. coli O157* ATCC35150; and (**F**): *E coli* ATCC10305. (1) 50 μg·mL^−1^ gentamicin; (2) rTurgencin A fermentation supernatant, the concentration of rTurgencin A in the fermentation supernatant was 9 μg·mL^−1^; and (3) laboratory fermentation supernatant of SC3 that did not express activity.

Inhibition profiling experiments were conducted to verify the activity of the rTurgencin A. The strains tested were Gram-positive bacteria, including *Bacillus subtilis* (*B. subtilis*) ATCC6633, *S. aureus* ATCC25923, and *Listeria monocytogenes* (*L. monocytogenes*) ATCC21633; and Gram-negative bacteria, including *Salmonella* spp. ATCC10467, *Escherichia coli* (*E. Coli*) O157 ATCC35150, and *E. coli* ATCC10305. The results showed that the rTurgencin A in this study provided antibacterial activity against Gram-positive and Gram-negative bacteria (Figure 3). In an agar well diffusion assay against *S. aureus* ATCC25923 and *L. monocytogenes* ATCC21633, 9 μg·mL^−1^ rTurgencin A fermentation supernatant was more significant than 50 μg·mL^−1^ gentamicin in terms of the inhibition circle diameter. It can be seen that the antibacterial effect of rTurgincin A was better than that of gentamicin. To more intuitively evaluate the antibacterial effect of rTurgincin A, its minimum inhibitory concentration (MIC) was tested (Table 2). The result showed that the MIC ranged from 2 to 5 μg·mL^−1^, and rTurgencin A showed overall inhibitory activity against Gram-positive and Gram-negative bacteria. In the study of Ida K. Ø. Hansen [28], the MIC of the natural antimicrobial peptide Turgencin A ranged from about 0.5 to 8.0 μg·mL^−1^, which is almost consistent with the MIC of the recombinant Turgincin A. The active fragments of Turgencin A have been synthesized chemically in the literature [29], with an MIC ranging from 1 to 62.5 μg·mL^−1^. The antimicrobial peptides expressed in *Pichia pastoris* may have higher antibacterial activity than chemically synthesized antimicrobial peptides. In addition, the chemical synthesis of antimicrobial peptides can sometimes render them inactive and expensive, making them unsuitable for large-scale use. The expression of antimicrobial peptides by *Pichia pastoris* has the potential for large-scale production, and our research provides the basis for its subsequent application.

### 2.4. Stability of Recombinant Turgencin A

**Figure 4:**
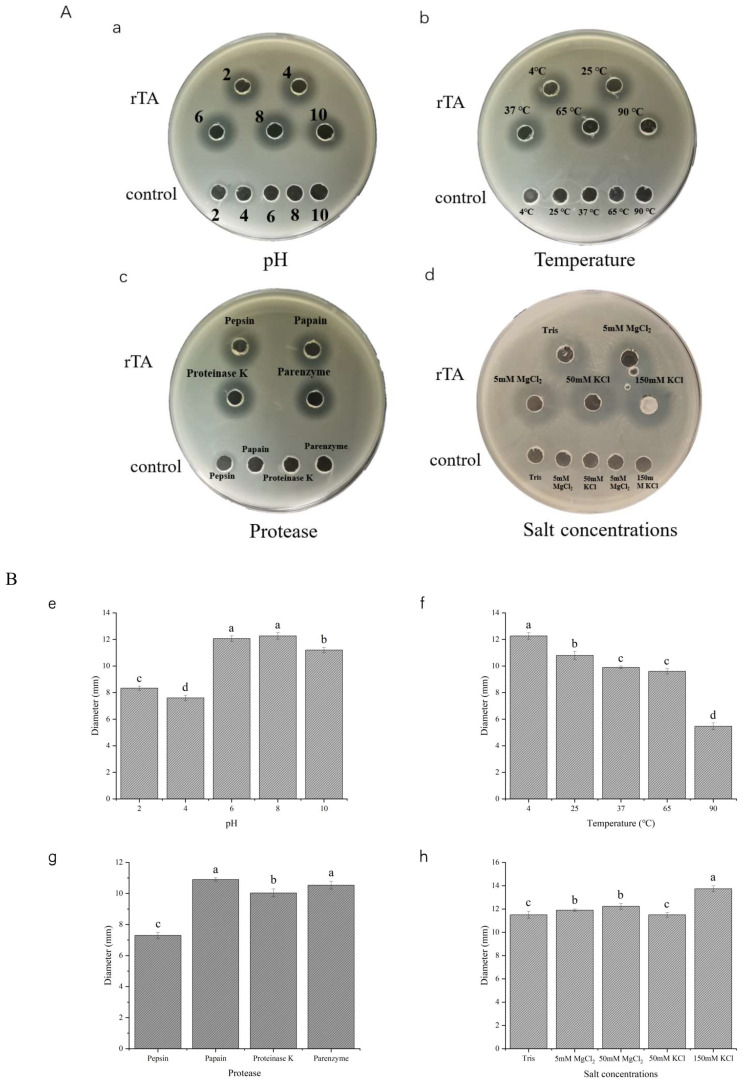
Turgencin A stability study: (**A**) Inhibition circle pictures corresponding to stability study. Negative control: (**a**) the buffer solution of different pHs, (**b**) the 50 mM pH 7.4 Tris-HCl solution treated at different temperatures, (**c**) the different protease solutions, and (**d**) the different salt solutions; (**B**) Histogram of stability study analysis, Y axis: diameter of inhibition circle. (**e**) Effect of pH on Turgencin A stability; (**f**) effect of temperature on Turgencin A stability; (**g**) effect of protease on Turgencin A stability; and (**h**) effect of salt concentration on Turgencin A stability. The same letters (a–d) indicate no significant difference between groups, and different letters indicate statistically significant differences between groups (*p* < 0.05) as calculated by one-way ANOVA followed by Tukey’s test.

To verify the stability of rTurgencin A, we tested its antimicrobial activity against *S. aureus* ATCC25923 by treating it at different temperatures and using different pHs, protease, and salt concentrations (Figure 4). In Figure 4(Aa), rTurgencin A was treated at the 2, 4, 6, 8, and 10 pH levels. The agar well diffusion assay revealed that rTurgencin A had a good stability at neutral and high pH levels and a 40% reduction in its inhibitory action at low pHs. Antibacterial peptides can achieve their best antibacterial effects only in a specific structural state, and their structural stability depends on pH [37]. Under the extreme conditions of strong acid, the structure of antibacterial peptides may change, thus affecting their antibacterial activity. In Figure 4(Ab), rTurgencin A showed a good stability at low and room temperatures of 4 °C, 25 °C, and 37 °C. However, after the high-temperature treatment at 90 °C for 1 h, the diameter of the inhibition circle was 55% of the maximum inhibition circle diameter, but it still had a good activity. Under different protease treatment concentrations, rTurgencin A was found to be reduced to 70% of its antibacterial activity in the presence of pepsin, but it showed a good stability in papain, k-protease, and trypsin (Figure 4(Ac)). The cleavage sites of pepsin are aromatic amino acids and acidic amino acids, while Turgencin A contains glutamic acid and tryptophan, which may reduce the activity of the peptide via enzymolysis. Furthermore, the antibacterial activity of rTurgencin A was not affected by salt solution concentrations of 5 mM MgCl_2_, 50 mM MgCl_2_, 50 mM KCl, and 150 mM KCl (Figure 4(Ad)). These results show that rTurgencin A has a good stability and inhibitory activity. This property offers the possibility of further applications of antimicrobial peptides for food preservation.

### 2.5. Mechanism Studies

We then conducted a total nucleotide leakage assay and used electron scanning microscopy and a cell permeation assay to investigate rTurgencin A’s inhibition mechanism.

#### 2.5.1. Scanning Electron Microscope

**Figure 5:**
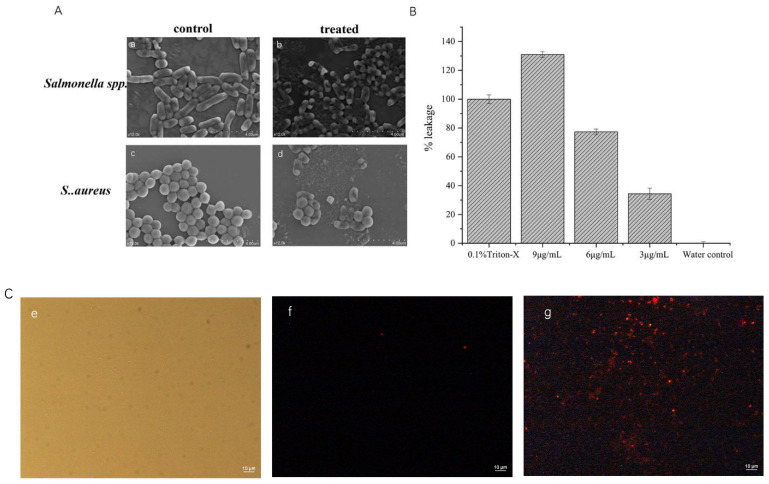
(**A**) (**a**). Control group: *Salmonella* spp. ATCC10467 without treatment with Turgencin A antimicrobial peptide; (**b**). Experimental group: *Salmonella* spp. ATCC10467 treated with Turgencin A antimicrobial peptide (10 μg·mL^−1^); (**c**). Control group: *S. aureus* ATCC25923 without treatment with Turgencin A antimicrobial peptide; and (**d**). Experimental group: *Salmonella* spp. ATCC10467 treated with Turgencin A antimicrobial peptide (10 μg·mL^−1^); (**B**) Total nucleotide leakage detected from *S. aureus* ATCC25923 after 4 h of inoculation and incubation; and (**C**) Fluorescence images of *S. aureus* ATCC25923 treated with PI: (**e**). Imaging of PBS-treated *S. aureus* ATCC25923 under white light; (**f**). Imaging of PBS-treated *S. aureus* ATCC25923 under fluorescence; and (**g**). Imaging of Turgencin A (10 μg·mL^−1^)-treated *S. aureus* ATCC25923 under fluorescence.

Scanning electron microscopy was used to observe the effect of rTurgencin A on the morphology of *S. aureus* ATCC25923 and *Salmonella* spp. ATCC10467 (Figure 5A). As shown in the figure, the surfaces of *S. aureus* ATCC25923 and *Salmonella* spp. ATCC10467 that were not treated with Turgencin A were smooth and round without wrinkles. However, the surfaces of *S. aureus* ATCC25923 and *Salmonella* spp. ATCC10467 treated with 10 μg·mL^−1^ of rTurgencin A were wrinkled and the cells appeared to be broken and ruptured, with leakage of the contents. These results suggest that the bacterial inhibition mechanism of rTurgencin A may involve cell death via cell membrane rupture.

In the study of Ida K. Ø. Hansen [29], the active fragment in the antimicrobial peptide Turgencin A was synthesized, and its mechanism of inhibition was investigated. Its active fragment could disrupt the cell membrane integrity of *B.* subtilis 168 (pCSS962) and *E. coli* K12 (pCSS962). In our study, rTurgencin A could disrupt the cell membranes of *S. aureus* ATCC25923 and *Salmonella spp*. ATCC10467. Both studies showed that Turgencin A can cause cell death by disrupting the integrity of cell membranes.

#### 2.5.2. Total Nucleotide Leakage

To further verify that Turgencin A disrupted the cell membrane of *S. aureus* ATCC25923, we conducted a total nucleotide leakage assay (Figure 5B). Using 0.1% Triton-X100 as a positive control, the OD was measured as 0.666 at 9 μg·mL^−1^ after a 4 h latency period when treated with different concentrations of rTurgencin A, whereas the OD value of the negative control using distilled water only was 0.149. The nucleotide leakage levels were related to the peptide concentration, with OD values of 0.453 and 0.284 corresponding to 6 μg·mL^−1^ and 3 μg·mL^−1^ of Turgencin A, respectively. At 1 × MIC, Turgencin A produced 34.3% of the total nucleotide leakage relative to the positive control; at 2 × MIC, Turgencin A produced 77.3% of the total nucleotide leakage; and at 3 × MIC, Turgencin A produced 131.5% of the total nucleotide leakage, exceeding the positive effect of the positive control. This indicates that rTurgencin A with 3 × MIC is powerful for cell membrane disruption.

#### 2.5.3. Cell Permeation Experiments

Propidium Iodide (PI) fluorescent dye was used to stain PBS and Turgencin-A-treated *S. aureus* ATCC25923. The PI dye could not cross intact cell membranes, but when these cell membranes were disrupted, PI entered the cells and bound to DNA, evidenced by the red. As shown in Figure 5C, the entire field of view of the control group was black, whereas the treated group contained evident red-colored bacteria. After an incubation of rTurgencin A with *S. aureus* ATCC25923 at 37 °C for 30 min, PI was able to penetrate the cytoplasmic membrane and bind to the bacterial nucleic acid; this produced the red fluorescence, indicating that most cells had a changed cell permeability. Therefore, we propose that rTurgencin A permeabilizes and damages the bacterial membrane of *S. aureus* ATCC25923, resulting in efficient bactericidal activity.

### 2.6. Hemolysis Assay

**Figure 6:**
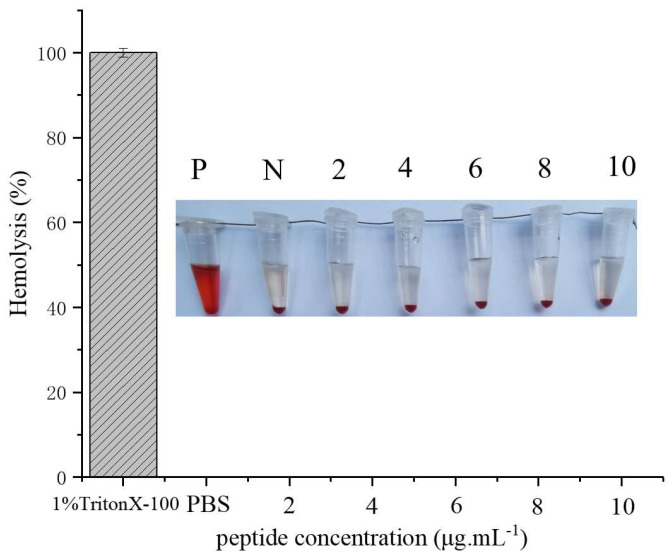
Hemolytic activity of the antimicrobial peptide Turgencin A on human erythrocytes. (P-Positive control, N-Negative control, peptide concentrations (μg·mL^−1^): 2, 4, 6, 8, and 10).

The hemolytic effect of Turgencin A peptide on human erythrocytes is shown in Figure 6. 1% Triton X-100 was used as the positive control for 100% hemolysis, and a PBS buffer was used as the negative control. rTurgencin A was not found to cause hemolysis, even at concentrations of 10 μg·mL^−1^. Our results suggest that the rTurgencin A peptide has a low hemolytic activity in human erythrocytes and is relatively safe for the human body. rTurgencin A has a high antibacterial and low hemolytic activity simultaneously, indicating that rTurgencin A could become an alternative antibiotic product.

### 2.7. Application of Turgencin A as Preservative

#### 2.7.1. Total Number of Bacteria

**Figure 7:**
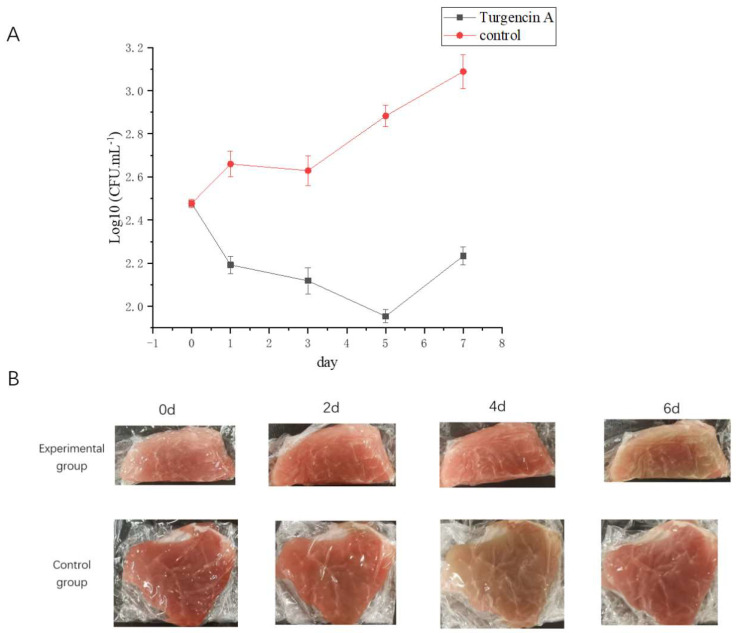
Analysis of the application of antimicrobial peptide Turgencin A in pork preservation: (**A**) Total number of bacteria in control and experimental groups (10 μg·mL^−1^) in pork on days 0, 1, 3, 5, and 7; and (**B**) Color change of pork on days 0, 2, 4, and 6.

Food corruption is mainly caused by bacterial action, and it is crucial to inhibit foodborne pathogens to extend the shelf life of food. Therefore, an antibacterial ability is one of the most essential characteristics of biological preservatives [38]. As shown in Figure 7A, the bacterial count of the control group showed an increasing trend with a change in pork storage days (0, 1, 3, and 5 days). In contrast, the bacterial colonies of the experimental group (pork treated with rTurgencin A at 10 μg·mL^−1^) showed a decreasing trend with an increase in days. The comparison between the experimental and control groups showed that the number of colonies in the experimental and control groups was similar on the first day. Still, as the days progressed, there were fewer colonies in the experimental group. The results showed that rTurgencin A inhibited the total food spoilage bacteria in pork and prolonged the storage time of the pork. Further study on its inhibitory effect on single bacteria could improve its application potential in food preservation.

#### 2.7.2. Flesh Color

The myoglobin in fresh meat maintains its reduced state, and its muscle color is burnt dark purple. When the ketone body is divided, with the contact between the muscle and air, the reduced state of myoglobin changes in two different directions. A part of the myoglobin and oxygen react to generate bright red, and another part of the myoglobin and oxygen react to generate brownish-brown. As the meat sits in the air for a long time, the meat color changes to maroon [39].

We observed the color of the pork flesh on days 0, 2, 4, and 6 (Figure 7B). It was found that, on day 4, the control group was already in a deteriorated state. The meat was brown and wrinkled on the surface. With the growth of the storage time, the meat color changed from brown to maroon; the meat of the experimental group (pork treated with rTurgencin A at 10 μg·mL^−1^) was still bright on the fourth day, and only on the sixth day did the meat of the experimental group appear dark brown. These results showed that rTurgencin A has a good preservation effect on pork. rTurgencin A delayed the time of pork spoilage by acting on the surface and inside of the pork. Based on maintaining the bright appearance of pork, its stored quality will be maintained for a longer time. Therefore, rTurgencin A has application potential as a pork preservative.

## 3. Materials and Methods

### 3.1. Strains and Plasmids

*S. aureus* ATCC25923, *Salmonella* spp. ATCC10467, *E. Coli* ATCC10305, *E. Coli* O157 ATCC35150, XL10 receptor, *L. monocytogenes* ATCC21633, *B. subtilis* ATCC6633, and GS115 were purchased from Pointe. The designed sequence of the Turgencin A plasmids was synthesized by Genewiz.

The amino acid sequence of Turgencin A is GPKTKAACKMACKLATCGKKPGGWKCKLCELGCDAV_._ The nucleic acid sequence of Turgencin A was optimized according to the codon preference of the *Pichia pastoris* expression system, and whole gene synthesis was conducted by Suzhou Genewiz. EcoRI cleavage sites, initiation codon ATG, 6 histidines, and the 15-base enterokinase site of “GATGATGATGATGATTAAG” were inserted at the N-terminus, termination codon TAA, and the XbaI cleavage sites were inserted at the C-terminus. The designed fragment was cloned into the pPICZαA vector between the corresponding restriction sites downstream of the α-factor signal peptide to realize the construction of the antibacterial peptide gene on the pPICZα A vector (Figure S2). The antimicrobial peptide was inserted into the pPICZα A vector at the digestion sites using EcoRI and XbaI restriction endonucleases obtained from LABLEAD Inc.

### 3.2. Structural Prediction

The secondary structure of Turgencin A was predicted by Webware [NPS@], provided by PRABI-Lyon-Gerland (https://npsa-prabi.ibcp.fr/cgi-bin/npsa_automat.pl?page=npsa_sopma.html) (accessed on 2 June 2023). We used swiss-model (https://swissmodel.expasy.org/interactive) (accessed on 2 June 2023) to predict Turgencin A’s tertiary structure. Then, we used Pymol software to further analyze the electrostatic potential on Turgencin A’s surface. In addition, we used the ProtScale tool in ExPASy (https://web.expasy.org/) (accessed on 2 June 2023) to analyze the hydrophobicity of Turgencin A.

### 3.3. Transformation of Pichia pastoris GS115

The synthesized plasmid was linearized with SacI restriction endonuclease (Beyotime, Shanghai, China). Then, the linear plasmid was transformed with GS115 Chemically Competitive Cell (Protein Interaction Bio. Co., Ltd., Wuhan, China), which we operated according to the manufacturer’s instructions. The obtained monoclonal transformants were activated on a Year Extract Peptone Dextrose (YPD) Petri dish containing 100 μg·mL^−1^ of zeocin. The genome of *Pichia pastoris* was extracted using a yeast DNA extraction kit (Thermofisher Scientific, Waltham, MA, USA). Then, specific primers were used for amplification to identify whether the Turgincin A gene had been successfully integrated into the genome of *Pichia pastoris*. The pPICZα A vector was used as the negative control. After PCR (5′-gcgaattcatgggttctcatcatcatcatcatcatgatgatgatgataa g-3′; 5′-gctctagattaacagcatcacaacccaattc-3′), the product was subjected to agarose gel electrophoresis, and the DNA was recovered using a Gel Extraction Kit (CWBIO, Taizhou, China). Then, we sent the samples to Genewiz (Suzhou, China) for sequencing and compared the results with the Turgencin A gene sequence to determine whether the gene had been transferred to *Pichia pastoris* GS115.

### 3.4. Induction of Expression and Detection of Peptides Obtained from Recombinant Pichia pastoris

Single yeast colonies were seeded in 25 mL of Buffered Glycerol-complex Medium (containing 2% peptone, 1% yeast extract, 10 mM potassium phosphate buffer (pH = 6.0), 1.34% yeast nitrogen base, 0.004% biotin, and 1% glycerol) and then incubated at 30 °C and 220 rpm until the OD_600_ value reached 8–10. Then, the culture was centrifuged at 3000 rpm for 5 min and the cell pellet was collected. The cell pellet was resuspended in 10 mL of sterile distilled water, centrifuged at 3000 rpm for 5 min, and collected again. We repeated the process twice. The yeasts were resuspended in 200 mL of Buffered Methanol-complex Medium and methanol was added to obtain a final methanol concentration of 1%. The mixture was incubated at 30 °C and 220 rpm. Methanol was added every 24 h to maintain a constant concentration. Daily samples were taken and centrifuged at 12,000 rpm for 5 min to collect the yeast fermentation supernatant.

Tricine-SDS-PAGE was performed, mixing 40 μL of the yeast fermentation supernatant and 10 μL of sample buffer containing 1 M Tris and 50% glycerol, boiling for 10 min before the gel loading. The voltage was adjusted to 100 V until the sample reached the bottom of the separation gel, lasting approximately 3–4 h. The gel was stained with a Protein Silver Stain Kit (CWBIO, Taizhou, China) to observe the protein expression trend.

### 3.5. Purification of Recombinant Antibacterial Peptides

For the affinity purification with a nickel column, the Ni NTA Beads 6FF filler (Smart-Lifesciences, Changzhou, China) was added to the column, and the filler was rinsed twice with distilled water to remove ethanol, followed by two rinses with binding buffer (50 mM Tris-HCl pH = 7.9, 500 mM NaCl, and 10 mM Imidazole). The fermentation supernatant was treated with a 0.45 μm membrane filter, added to the nickel column, and combined with filler for 1 h in a low-speed shaker at 4 °C. The flow solution was collected and rinsed with binding buffer (50 mM Tris-HCl pH = 7.9, 500 mM NaCl, and 10 mM Imidazole) three times to wash off unbound proteins. Elution buffer (50 mM Tris-HCl pH = 7.9, 500 mM NaCl, and 1 M imidazole) was added and the eluent of the target protein was collected. Finally, the eluent was dialyzed in 0.01 M PBS for 12 h at a low temperature.

### 3.6. Determination of Turgencin A Concentration: ELISA

Indirect ELISA was used to detect the concentration of rTurgencin A [40]. The standard protein GFP (6his-tag) was continuously diluted with the coating solution (15 mM Na_2_CO_3_, 36 mM NaHCO_3_, and pH 9.8). In total, 200 μL of the standard and sample protein diluent was coated in a 96-well enzyme-labeled plate, with three repeated wells, and incubated at 4 °C overnight. Then, the plate was washed three times with 200 μL of PBST per well. A total of 200 μL of PBST containing 5% skim milk was added to each well, with slight shaking at 37 °C for 1 h, and the plate was washed three times. In total, 100 μL of Anti His-Tag Mouse MAb (1:2000 diluted in PBST) was added to each well, incubated at 37 °C for 1.5 h, and the plate was washed three times. A total of 100μL of HRP-Goat-anti-mouse secondary antibody (1:5000 diluted in PBST) was added to each well, incubated at 37 °C for 1.5 h, and the plate was washed three times. Then, 150 μL of TMB coloring buffer was added to each well and developed at room temperature in the dark for 30 min. Finally, 50 μL of 0.5M H_2_SO_4_ was added into each well to terminate the reaction. The optical density was read with a microplate ELISA reader at 450 nm. A standard curve was drawn between the concentration and absorbance of the standard solution. The absorbance value of the sample was substituted into the standard curve formula to obtain the concentration of rTurgencin A.

### 3.7. Antibacterial Activity

#### 3.7.1. Agar Well Diffusion Assay

The test bacterial strain was seeded into an LB liquid medium and cultured at 37 °C until the OD_600_ value reached 1. In total, 50 μL of bacteria was mixed with the 15 mL molten LB medium (1% peptone, 1% NaCl, and 0.5% yeast extract) at 25 °C and allowed to solidify. Holes were punched with sterile gun heads. Subsequently, 50 μL of the fermentation supernatant was added to the holes. Gentamicin (Gen) at 50 µg·mL^−1^ was used as the positive control. The antibacterial peptide SC3, which was not active in the laboratory, was used as the negative control.

#### 3.7.2. Minimum Inhibitory Concentration (MIC) Determination

A single colony of the tested bacteria was picked to LB liquid medium and incubated at 37 °C until the OD600 reached about 1. This test bacteria solution was diluted 1000 times. The fermentation supernatant was serially diluted in a 96-well plate using replacement buffer to 1, 2, 3, 4, 5, and 6 μg·mL^−1^ concentrations. Then, 20 μL of the diluted peptide and 100 μL of the test bacterium were added to each well. The negative control and positive control were 50 μg·mL^−1^ of gentamicin and the replacement buffer, respectively. The MIC value was defined as the lowest peptide concentration associated with no bacterial growth. In this study, the MIC values of Turgencin A were determined against *S. aureus* ATCC25923, *Salmonella* spp. ATCC10467, *E. coli* ATCC10305, *E. Coli* O157 ATCC35150, *L. monocytogenes* ATCC21633, and *B. subtilis* ATCC6633.

### 3.8. Stability Study of Recombinant Antimicrobial Peptides

The rTurgencin A freeze-dried fermentation supernatant was dissolved in different buffers. The contents were all 200 mg·mL^−1^. Therefore, the concentrations of rTurgencin A in this experiment were all 13.7 μg·mL^−1^.

#### 3.8.1. Study of Temperature Stability

The rTurgencin A freeze-dried fermentation supernatant was dissolved in a 50 mM pH 7.4 Tris-HCl solution and then treated at 4 °C, 25 °C, 37 °C, 65 °C, and 90 °C for 1 h. The samples were subjected to an agar well diffusion assay. Twelve hours later, the inhibition circle was observed. The negative control was a 50 mM pH 7.4 Tris-HCl solution after treatment for 1 h at the same temperature.

#### 3.8.2. Study of pH Stability

The rTurgencin A freeze-dried fermentation supernatant was dissolved in a glycine solution at a pH of 2, a sodium acetate solution at a pH of 4, a Tris-HCl solution at a pH of 6, a Tris-HCl solution at a pH of 8, and a glycine solution at a pH of 10. The final concentrations of the buffer solution were all 100 mM. Then, the samples were treated at 37 °C for 4 h and subjected to the agar well diffusion assay. Twelve hours later, the inhibition circle was observed. The negative control was a buffer solution of different pHs.

#### 3.8.3. Study of Protease Stability

The rTurgencin A freeze-dried fermentation supernatant was dissolved in buffers of a glycine solution at a pH of 2, a PBS solution at a pH of 6, a Tris-HCl solution at a pH of 7, and a Tris-HCl solution at a pH of 8. The final concentrations of the buffer solution were all 100 mM. Pepsin was added to the solution at a pH of 2, papain at a pH of 6, proteinase K at a pH of 7, and trypsin at a pH of 8. The protease concentrations were all 1 mg·mL^−1^. The mixtures of 0.24 U of different protease solutions and rTurgencin A were reacted at 37 °C for 2 h. The samples were then subjected to an agar well diffusion assay. Twelve hours later, the inhibition circle was observed. The negative control was different protease solutions.

#### 3.8.4. Study on the Stability of Salt Concentration

The rTurgencin A freeze-dried fermentation supernatant was dissolved in 5 mM MgCl2, 50 mM MgCl2, 50 mM KCl, and 150 mM KCl, respectively. Then, the samples were subjected to an agar well diffusion assay. Twelve hours later, the inhibition circle was observed. The negative control was the different salt solutions.

### 3.9. Effect of Turgencin A on Cell Membrane

#### 3.9.1. Total Nucleotide Leakage

The total nucleotide leakage associated with the peptide activity was analyzed according to the protocol described by Shwaiki et al. [41]. We detected the total nucleotide leakage of the effect of Turgencin A on the cell membrane of *S. aureus* ATCC25923. The OD of each concentration was measured at 260 nm after four hours of contact reaction. The positive and negative controls were 0.1% Triton X-100 and water, respectively.

#### 3.9.2. Scanning Electron Microscopy Observations

Single colonies of *S. aureus* ATCC25923 and *Salmonella* spp. ATCC10467 were cultured in LB liquid medium to a logarithmic growth period at 37 °C. They were centrifuged at 3000 rpm for 5 min and the supernatant was discarded. The cell pellet was suspended in sterile water, centrifuged, and the supernatant was discarded; this was repeated three times, and then the cell pellet was resuspended to 1 × 10^8^ CFU·mL^−1^. Subsequently, 100 μL of a bacterial solution and 50 μL of 10 μg·mL^−1^ of Turgencin A were incubated at 37 °C for 30 min, and Phosphate-Buffered Saline (PBS) was used as a negative control. The supernatant was discarded after centrifugation and the cell pellet was resuspended with sterile water. The sample was dropped on the coverslips, dried, and submerged with 2.5% glutaraldehyde for 40–50 min. Then, the glutaraldehyde was washed off with 0.1 mol·L^−1^ of phosphate buffer. Ethanol solution (30%, 50%, and 70%) was used to dehydrate for 10 min, respectively. After drying, the morphology of the bacteria was observed using a scanning electron microscope (SEM).

#### 3.9.3. Cell Permeability Assay

Single colonies of *S. aureus* ATCC25923 were cultured in LB liquid medium to a logarithmic growth period at 37 °C. They were centrifuged at 3000 rpm for 5 min and the supernatant was discarded. The cell pellet was suspended in sterile water, centrifuged, and the supernatant was discarded; this was repeated three times, and then the cell pellet was resuspended to 1 × 10^8^ CFU·mL^−1^. Subsequently, 100 μL of a bacterial solution and 50 μL of 10 μg·mL^−1^ of Turgencin A were incubated at 37 °C for 30 min, and PBS was used as a negative control. The supernatant was discarded after centrifugation, and the cell pellet was resuspended in 1:3000 diluted propidium iodide. This was stained in the dark at 37 °C for 30 min. The supernatant was discarded after centrifugation, and the cell pellet was resuspended in sterile water, with this being repeated three times. A proper amount of samples was added and we observed them under a fluorescence microscope.

### 3.10. Hemolytic Analysis of Turgencin A

Fresh human blood was collected and processed using centrifugation at 1500 rpm for 10 min to remove serum. The blood cells were washed three times with PBS, resuspended in PBS, and diluted to a cell concentration of 4% (*v*/*v*). Subsequently, 10 μL of antimicrobial with concentrations of 2, 4, 6, 8, and 10 μg·mL^−1^ were mixed with 90 μL of the blood cell solution, incubated at 37 °C for 1 h, centrifuged at 1500 rpm for 10 min, and the absorbance at OD_576_ was then measured. 1% Triton X-100 was used as the positive control and a PBS buffer was used as the negative control.

### 3.11. Pork Preservation Experiment

The fermentation supernatant was passed through a 0.22 μm membrane filter and transferred to a beaker. Fresh pork hind leg meat was cut into 5 g pieces and placed in the above solution, soaked for 1 min in each direction, shaken so that the surface of the meat was fully exposed to the solution, removed, placed on cling film to drain, left for 5 min at room temperature, turned over, and left for another 5 min to remove excess water so that the meat neither dripped nor was too dry. The meat was then wrapped in plastic and stored in a refrigerator at 4 °C for seven days. During this period, the pork samples were tested for colony counts every 48 h, according to the method of GB 4789-2022 [42]. The colony count is expressed by colony-forming units (CFU), and a common logarithm determines the total number of colonies. SC3 served as the negative control.

### 3.12. Statistical Analysis

Excel 2019 was used for data recording and sorting. All the data were analyzed using SPSS 29 for an analysis of variance (ANOVA) test. The significance level was set to *p* < 0.05. The Duncan test was used to compare pairs of means. The experimental results were the averages of three experiments (*n* = 3).

## 4. Conclusions

*S. aureus* is a common food-borne microorganism and the overuse of antibiotics has increased its bacterial resistance. Therefore, it is very crucial to develop new green preservatives. This study illustrates that the recombinant antimicrobial peptide expressed by *Pichia pastoris* has potential as a new food preservative. We efficiently expressed the antimicrobial peptide Turgencin A using a recombinant expression method and studied its biological characteristics.

The structure prediction results showed that Turgencin A is an α-helix cationic peptide with three disulfide bonds, which play an essential role in the antibacterial activity of Turgencin A. Studies have shown that disulfide bonds in peptides have a critical function for the antibacterial activity of peptides. Antibacterial peptides can achieve their best antibacterial effect only in a specific structural state [37]. The MIC of naturally obtained Turgencin A ranges from 0.5 to 8.0 μg·mL^−1^ [28]. The MIC of the rTurgencin A expressed by *Pichia pastoris* ranges from 2 to 5 μg·mL^−1^, and the MIC of an active fragment of chemically synthesized Turgencin A ranges from 1 to 62.5 μg·mL^−1^ [29], which indicates that rTurgencin A has good antibacterial activity. Therefore, we speculate that the mode of the intramolecular disulfide bond in rTurgencin A should be the same as that in natural Turgencin A.

We constructed a positive yeast strain that can efficiently express Turgencin A so that Turgencin A can be secreted and expressed in a yeast fermentation supernatant. rTurgencin A has a 6his-tag, so we hoped to obtain a high-purity antibacterial peptide product of rTurgencin A using nickel column affinity chromatography. However, the purified product of rTurgencin A had deficient antibacterial activity after the dialysis of the replacement solution, so we chose to contain the yeast fermentation supernatant of rTurgencin A in the follow-up experiments and determine the concentration of the target peptide rTurgencin A in the supernatant using the ELISA method.

The results of further research show that rTurgencin A has broad-spectrum antibacterial activity, a good stability, and low hemolytic activity, which indicates that rTurgencin A has good application potential. In addition, we studied the inhibition mechanism of rTurgencin A. It was determined that rTurgencin A can destroy the cell membrane of bacteria, make the cell contents leak, and then kill the bacteria. We also performed a preliminary study on its application in food preservation. We found that rTurgencin A has a significant food preservation effect because it can inhibit the growth of bacteria, thus maintaining the original color of pork for a longer time. However, it is necessary to analyze the immunogenicity of rTurgencin A. These experimental results provide primary data for the application prospects of rTurgencin A.

At present, we still face many problems. The yield of the nickel column purification was low, and the antibacterial activity of the purified product was weak. It is necessary to explore the purification conditions further. When producing antibacterial peptides on a large scale, it is necessary to deodorize them to apply them to different fields.

## Figures and Tables

**Table 1:** Detection of antimicrobial peptide concentrations using ELISA.

Peptide	Concentration (μg·mL^−1^)
Turgencin A	11.23
200 mg·mL^−1^ TA fermentation supernatant freeze-dried	13.71

**Table 2:** Minimum inhibitory concentration (MIC) of Turgencin A.

Bacteria Strain	MIC (μg·mL^−1^)
Gram−positive	
*S. aureus* (ATCC 25923)	3
*B. subtilis* (ATCC 6633)	2
*L. monocytogenes* (ATCC 21633)	3
Gram−negative	
*Salmonella* spp. (ATCC 10467)	4
*E. coli O157* (ATCC 35150)	5
*E. coli* (ATCC 10305)	5

## Data Availability

Data is contained within the article or Supplementary Materials.

## Supplementary Materials

The following supporting information can be downloaded at: https://www.mdpi.com/article/10.3390/molecules28145405/s1, Figure S1: Sequencing results; Figure S2: Comparison of sequencing results with Turgencin A gene sequences; Figure S3: Plasmid Mapping.

## Sample Availability

Samples of the compounds are available from the authors.
