# Supplementary material for: The Expression of Antibacterial Peptide Turgencin A in Pichia pastoris and an Analysis of Its Antibacterial Activity
Source: Molecules. 2023 Jul 14;28(14):5405. doi: 10.3390/molecules28145405 (PMC10384874; doi:10.3390/molecules28145405)

supplemental material

Figure S1. Sequencing results:

ATTTTCCAACAGCACAAATAACGGGTTATTGTTTATAAATACTACTATTGCCAGCATTGC
TGCTAAAGAAGAAGGGGTATCTCTCGAGAAAAGAGAGGCTGAAGCTGAATTCATGGG
TTCTCATCATCATCATCATCATGATGATGATGATAAGGGTCCAAAGACTAAGGCTGCTTG
TAAGATGGCTTGTAAGTTGGCTACTTGTGGTAAGAAGCCAGGTGGTTGGAAGTGTAAG
TTGTGTGAATTGGGTTGTGATGCTGTTTAATCTAGAACAAAAACTCATCTCAGAAGAGG
ATCTGAATAGCGCCGTCGACCATCATCATCATCATCATTGAGTTTGTAGCCTTAGACATG
ACTGTTCCTCAGTTCAAGTTGGGCACTTACGAGAAGACCGGTCTTGCTAGATTCTAATC
AAGAGGATGTCAGAATGCCATTTGCCTGAGAGATGCAGGCTTCATTTTTGATACTTTTT
TATTTGTAACCTATATAGTATAGGATTTTTTTTGTCATTTTGTTTCTTCTCGTACGAGCTTG
CTCCTGATCAGCCTATCTCGCAGCTGATGAATATCTTGTGGTAGGGGTTTGGGAAAATC
ATTCGAGTTTGATGTTTTTCTTGGTATTTCCCACTCCTCTTCAGAGTACAGAAGATTAAG
TGAGACCTTCG

Figure S2. Comparison of sequencing results with Turgencin A gene sequences

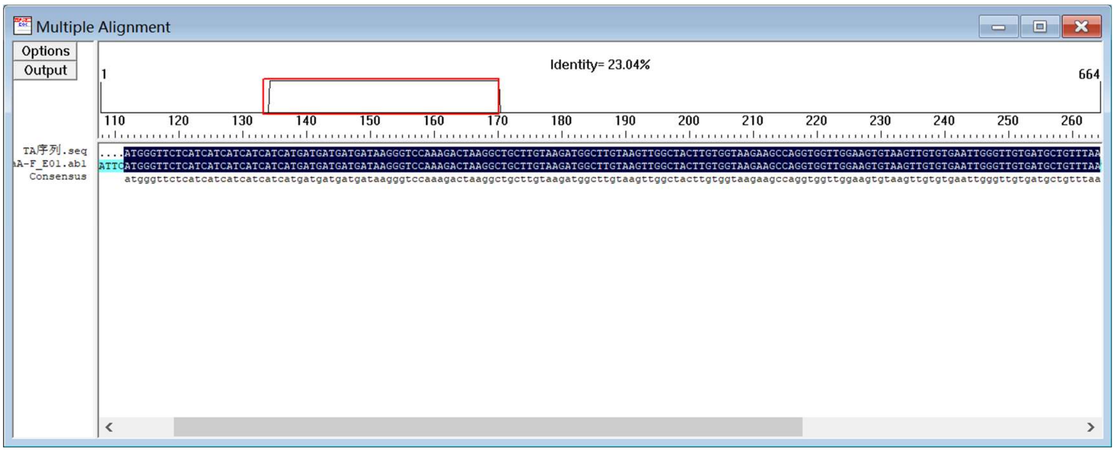

Figure S3. Plasmid Mapping:

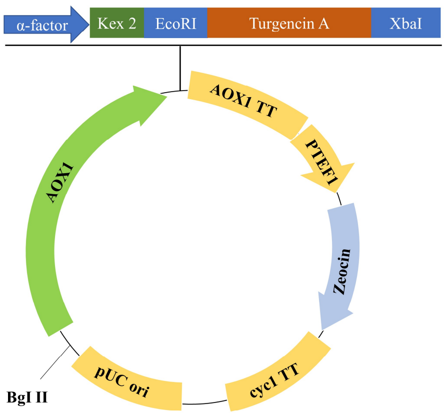

Supplement: Supplementary file 1 [file molecules-2465937-supplementary.pdf › molecules-2465937-supplementary.pdf]
